# The treatment of myocardial ischemia-reperfusion injury: potential roles of natural drug modulating CaMKII

**DOI:** 10.3389/fphar.2025.1718008

**Published:** 2026-01-14

**Authors:** Kai Yang, Ping Zhang, Xianshan Hui, Chao Liu, Jun Li, Yongmei Liu

**Affiliations:** 1 Guang’anmen Hospital, China Academy of Chinese Medical Sciences, Beijing, China; 2 Shandong University of Traditional Chinese Medicine, Jinan, China

**Keywords:** anti-inflammatory, CaMKII signaling pathway, myocardial ischemia-reperfusion injury, natural drugs, phytochemicals, systematic review

## Abstract

Myocardial ischemia-reperfusion injury (MIRI) represents a significant challenge in the treatment of cardiovascular diseases, with its complex pathogenesis involving multiple signaling pathways, such as calcium overload, oxidative stress, inflammatory responses, and cell apoptosis. Calcium/calm-odulin-dependent protein kinase II (CaMKII), a key calcium signaling transducer molecule, plays a central regulatory role in the onset and progression of MIRI. We conducted a comprehensive review of the literature on phytochemicals targeting CaMKII as a protective mechanism against MIRI. The search was performed across multiple databases, including PubMed, Web of Science, China National Knowledge Infrastructure (CNKI), and Google Scholar, covering the period from January 2000 to September 2025. A total of 254 articles were retrieved, of which 16 were included in this review. Molecular docking was then performed to evaluate the binding affinity between natural drugs and CaMKII. This paper systematically summarizes the latest research findings on natural drugs that alleviate MIRI by targeting the CaMKII signaling pathway, with a particular focus on the mechanisms of action of representative natural drugs and their experimental validation in animal and cellular models. The aim is to provide a theoretical basis and research directions for the development and clinical application of natural drugs, thereby promoting innovation in prevention and treatment strategies for myocardial ischemia-reperfusion injury.

## Graphical Abstract

**Figure:**
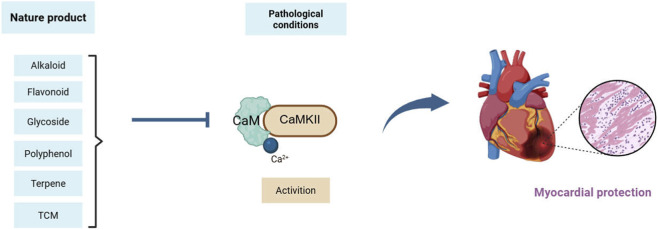

## 1 Introduction

Myocardial ischemia-reperfusion injury (MIRI) refers to the further damage to myocardial cells that occurs when blood flow is restored after a brief period of ischemia in the heart. This pathological process is complex and involves multiple mechanisms, including calcium ion homeostasis imbalance, excessive generation of reactive oxygen species (ROS), activation of inflammatory responses, mitochondrial dysfunction, and apoptosis. Abnormal regulation of calcium ion signaling plays a central role in MIRI. In particular, calcium/calmodulin-dependent protein kinase II (CaMKII), an important signal transduction molecule in myocardial cells, can sense changes in calcium signals and regulate myocardial cell contractile function, metabolic activity, apoptosis, and inflammatory responses. Numerous studies have demonstrated that the excessive activation of CaMKII is a critical link in MIRI damage, making it a potential therapeutic target (Yao et al., 2022; Zhang et al., 2023).

The predominant subtype of CaMKII in the myocardium is CaMKII-δ, which exerts different biological functions through multiple splice variants. For example, CaMKII-δ9 and CaMKII-δ3, as the main splice isoforms, exhibit opposing effects in regulating myocardial cell survival: δ9 promotes cell death, whereas δ3 has a protective effect. Specific inhibition of CaMKII-δ9 has been shown to effectively reduce cardiac damage and inflammatory responses caused by myocardial ischemia-reperfusion, significantly improving myocardial remodeling and the occurrence of heart failure (Yao et al., 2022). This finding not only reveals a new mechanism by which CaMKII-δ9 regulates myocardial inflammation through direct interaction with IκBα, a key metabolite of the NF-κB signaling pathway, but also provides a novel targeted strategy for the treatment of myocardial ischemia-reperfusion injury.

During the process of MIRI, calcium ion overload is a significant cause of mitochondrial dysfunction and myocardial cell apoptosis. Research has shown that the expression of the mechanosensitive cation channel Piezo1 is elevated in myocardial ischemia-reperfusion regions. Its deletion can improve mitochondrial function, reduce inflammation and oxidative stress, alleviate calcium ion overload, and thereby mitigate myocardial damage by regulating mitochondrial fusion and fission (Xu et al., 2025). Additionally, long-chain acylcarnitines, such as palmitoylcarnitine, can induce intracellular calcium ion accumulation, disrupt mitochondrial membrane potential, activate the mitochondrial permeability transition pore (mPTP), and promote cell death, suggesting a close relationship between calcium homeostasis disturbances and mitochondrial energy metabolism collapse (Berezhnov et al., 2020). As the center of cellular energy metabolism, mitochondrial dynamic balance (fusion and fission) and functional maintenance are crucial for the survival of myocardial cells. Natural drugs like Flavagline3 have been shown to significantly reduce myocardial cell apoptosis and cardiac dysfunction by promoting mitochondrial fusion and enhancing mitochondrial-endoplasmic reticulum (ER) interactions, thereby regulating calcium ion homeostasis and demonstrating the potential to regulate mitochondrial dynamics and calcium signaling (Zhong et al., 2025). Similarly, Sirtuin family members SIRT1 and SIRT3 play critical roles in maintaining mitochondrial homeostasis and myocardial contractile function. Their deletion leads to abnormal calcium ion flux and impaired mitochondrial respiratory function, exacerbating ischemia-reperfusion injury (Zhang et al., 2021a).

The activation of CaMKII not only promotes myocardial cell apoptosis but also participates in the regulation of inflammatory signaling pathways. The CaMKII-δ9 subtype promotes IκBα phosphorylation by directly interacting with IκBα, which inhibits NF-κB, thereby activating the NF-κB signaling pathway and exacerbating myocardial inflammatory responses and damage (Yao et al., 2022). Furthermore, abnormal activation of CaMKII in viral myocarditis also promotes the release of inflammatory factors and myocardial cell death. The natural drug Danhong Injection, through its active metabolite dihydrotanshinone I, inhibits CaMKII, reduces myocardial cell apoptosis and inflammation, and shows promising therapeutic prospects (Zhang et al., 2025).

Due to their advantages of multi-target regulation and low toxicity, natural drugs have become an important direction for regulating the CaMKII signaling pathway to intervene in MIRI. Dihydrotanshinone I, an active metabolite in Danhong Injection, acts as a CaMKII inhibitor and reduces myocardial cell death and inflammation associated with viral myocarditis (Zhang et al., 2025). Additionally, natural products such as flavonoids and saponins improve mitochondrial function, inhibit oxidative stress and inflammatory responses, and exert myocardial protective effects by regulating CaMKII and its downstream signals (Wang et al., 2021a; Sharma et al., 2024). These studies provide a theoretical and experimental basis for targeting the CaMKII signaling pathway with natural drugs to treat myocardial ischemia-reperfusion injury.

In summary, the occurrence and development of myocardial ischemia-reperfusion injury are closely related to calcium ion homeostasis imbalance, mitochondrial dysfunction, oxidative stress, and inflammatory responses. As a key regulator of calcium signaling, CaMKII plays a central role in myocardial cell apoptosis and inflammatory responses. Natural drugs, through multi-target regulation of the CaMKII signaling pathway, improve mitochondrial function, inhibit oxidative stress and inflammatory responses, and demonstrate promising myocardial protective potential. Future in-depth exploration of the molecular mechanisms by which natural drugs regulate the CaMKII signaling pathway will provide new strategies and directions for drug development in the prevention and treatment of myocardial ischemia-reperfusion injury.

## 2 Structure and activation mechanism of CaMKII

### 2.1 Structure of CaMKII

**FIGURE 1:**
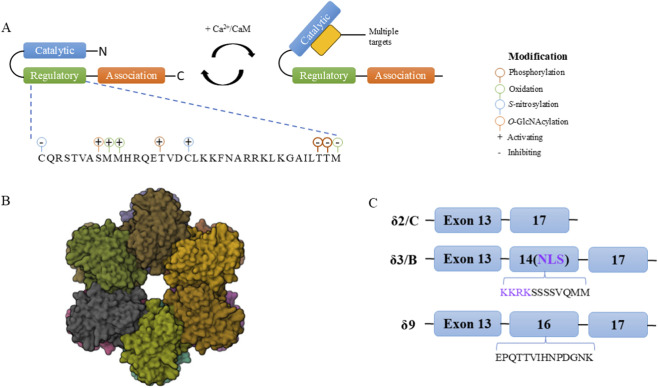
**(A)** The schematic diagram of the CaMKII monomer shows that upon binding to Ca^2+^/calmodulin (CaM), the catalytic domain is released from its autoinhibitory pseudosubstrate and becomes capable of phosphorylating its downstream targets. Several post-translational modifications in the regulatory domain positively and negatively regulate CaMKII activity. The panel is adapted from an image created using Biorender.com. **(B)** In the structural description of the CaMKII dodecameric holoenzyme, each domain is color-coded as in **(A)**. The structure (PDB ID: 3SOA) is sourced from the RCSB Protein Data Bank (rcsb.org) (CC0 1.0). **(C)** CaMKII δ undergoes alternative splicing to generate three prevalent variants in the heart. These variants differ only at the variable linker region through the inclusion or omission of exon 14 or 16.

CaMKII is a serine/threonine protein kinase composed of multiple subunits and is widely present in various human tissues. It mainly includes four subtypes: α, β, γ, and δ, with certain differences in structure and function among different subtypes. In particular, the CaMKIIδ subtype is the most abundantly expressed in myocardial tissue and undertakes multiple crucial functions within myocardial cells. CaMKII typically exists in the form of a dodecameric or tetradecameric holoenzyme, composed of multiple catalytic subunits. These subunits are interconnected through a central “hub” structure, forming a stable oligomeric structure (Karandur et al., 2020). Each subunit of CaMKII contains a catalytic domain, a regulatory segment, and a regulatory region that binds to calcium^2+^/calmodulin (CaM) (Nguyen et al., 2022). The binding of the calcium^2+^/CaM complex is the primary step in CaMKII activation. After binding, the kinase’s self-inhibited state is relieved, the catalytic domain is exposed, and the kinase activity is activated (Figure 1).

### 2.2 Activation mechanism of CaMKII

#### 2.2.1 Autophosphorylation ability

In the activation mechanism, CaMKII can achieve a sustained activated state through autophosphorylation (e.g., at the T286 site), allowing the kinase to remain active even after the dissociation of the calcium^2+^/CaM complex. This autophosphorylation endows CaMKII with “autonomous” activity, enabling it to continue executing downstream signal transduction functions after the calcium^2+^ signal has subsided (Giese, 2021). Relevant studies, through molecular simulations and experimental validation, have found that the binding of calcium^2+^/CaM not only activates CaMKII but also stabilizes the autophosphorylated state, preventing dephosphorylation mediated by protein phosphatases and thus prolonging kinase activity (Pharris et al., 2019). Additionally, the reciprocal activation complex (RAKEC) formed between CaMKII and the regulatory protein Tiam1 further supports the persistence of autophosphorylation-mediated activation. This mechanism is of great significance for long-term signal maintenance and structural plasticity (Saneyoshi et al., 2019; Saneyoshi, 2021).

#### 2.2.2 Oxidative modification

Oxidative modification is also an important way to activate CaMKII, especially under oxidative stress conditions such as myocardial ischemia-reperfusion injury. The oxidation of cysteine residues (e.g., Met281/282) leads to the sustained activation of CaMKII, forming oxidized CaMKII (ox-CaMKII). This oxidative modification allows CaMKII to remain active in the absence of calcium^2+^ activation, promoting calcium homeostasis disturbances and myocardial damage (Qu et al., 2019; Mesubi et al., 2021). Clinical and animal model studies have demonstrated that ox-CaMKII is an important pathogenic factor in myocardial pathological states, and inhibiting its oxidative activation can alleviate myocardial ischemia injury and arrhythmias (Mesubi et al., 2021; Qi et al., 2022).

During ischemia-reperfusion, the intracellular calcium^2+^ load in myocardial cells increases significantly, leading to the massive formation of calcium^2+^/CaM complexes, which further activate CaMKII. Meanwhile, the oxidative stress induced by ischemia-reperfusion promotes the abnormal activation of CaMKII through oxidative modification, resulting in a sustained active state and participating in the signal transduction of myocardial cell damage (Rocco-Machado et al., 2022; Zhong et al., 2025). In addition, the activation of CaMKII also promotes its interaction with multiple target proteins, such as the NMDAR subunit GluN2B, regulating ion channel function and cytoskeletal remodeling. These processes play key roles in myocardial cell damage and repair (Hosokawa et al., 2021; Rumian et al., 2024).

#### 2.2.3 Regulation by other signal molecules

It is worth mentioning that the activation of CaMKII not only depends on the binding of calcium^2+^/CaM but is also influenced by the interaction between its regulatory segment and other proteins. For example, cellular retinoic acid-binding protein 1 (CRABP1) inhibits the activation of CaMKII by preferentially binding to its inactive conformation through interaction with the CaMKII regulatory segment (Nhieu et al., 2023). This regulatory mechanism reveals a multi-level regulatory network for CaMKII activation.

In general, the activation of CaMKII is a multi-level and multi-mechanism process. Based on the classic calcium^2+^/calmodulin-dependent activation, autophosphorylation and oxidative modification provide guarantees for its sustained activity. These mechanisms collectively ensure that CaMKII plays a key role under pathological conditions such as myocardial ischemia-reperfusion.

## 3 Mechanisms of CaMKII-mediated myocardial cell injury

### 3.1 Regulation of myocardial cell apoptosis

**FIGURE 2:**
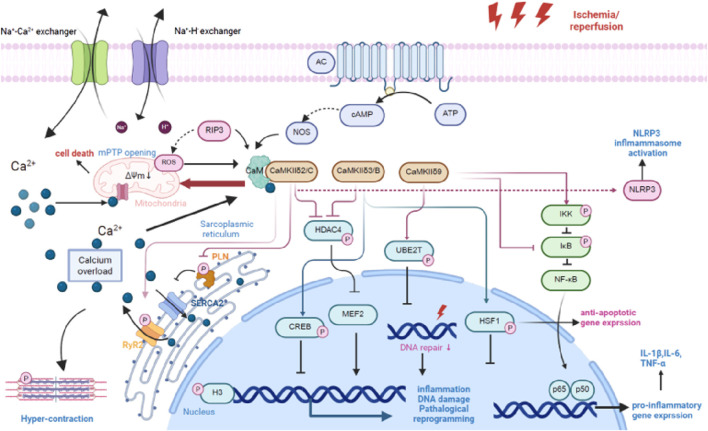
The role of CaMKII in MIRI. The role of CaMKII in MIRI begins with Ca^2+^endocytosis. Major known CaMKII signaling pathways in cardiomyocytes. Black arrows denote upstream stimulants, and faded arrows denote downstream pathological (red) and cardioprotective (blue) targets. Red phosphate groups denote phosphorylation by CaMKII. When known, arrow origin denotes the responsible splice variant. Abbreviations: CaMKII, multifunctional Ca^2+^ and calmodulin-dependent protein kinase II; CREB, cAMP response element–binding protein; H3, histone 3; HDAC4, histone deacetylase 4; HSF1, heat shock factor 1; I, current; IκB, inhibitor of nuclear factor kappa-B; IKK, inhibitor of nuclear factor kappa-B kinase; MEF2, myocyte-enhancer factor 2; NF-κB, nuclear factor kappa-B; NOX, nicotinamide adenine dinucleotide phosphate oxidase; PLN, phospholamban; ROS, reactive oxygen species; RyR2, type 2 ryanodine receptor; SERCA2, sarco/endoplasmic reticulum calcium ATPase 2; RIP3,Receptor Interacting Serine/Threonine Kinase 3; UBE2T, ubiquitin-conjugating enzyme E2 T. ROS, Reactive Oxygen Species; AC, Adenylate Cyclase; NOS, Nitric Oxide Synthase; NLRP3, NLR Family Pyrin Domain Containing 3. Figure adapted from image created with Biorender.com.

CaMKII plays a crucial role in MIRI by regulating myocardial cell apoptosis. The specific mechanism involves the phosphorylation regulation of multiple apoptosis-related target proteins by CaMKII, such as the pro-apoptotic proteins Bad and Bax, thereby activating the cell apoptosis signaling pathway and promoting programmed cell death in myocardial cells. After ischemia-reperfusion, the activation of CaMKII not only regulates apoptotic proteins through phosphorylation but also activates the mitochondrial pathway, inducing the release of cytochrome C and further exacerbating the cell apoptosis process. CaMKII interacts with calmodulin to regulate intracellular calcium homeostasis. Its overactivation leads to calcium overload, which induces mitochondrial dysfunction and promotes apoptosis (Jiang et al., 2023). In experiments, inhibiting CaMKII activity (e.g., using KN-93) significantly reduces myocardial cell apoptosis after ischemia-reperfusion and improves cardiac function (Kong et al., 2019). CaMKII also affects autophagy function and indirectly regulates apoptosis levels by participating in the phosphorylation regulation of the autophagy-related protein Beclin-1 (Kong et al., 2020). In summary, CaMKII mediates myocardial cell apoptosis through multiple signaling pathways and is an important regulatory factor in MIRI, making it a potential therapeutic target (Figure 2).

### 3.2 Mediation of inflammatory responses

The activation of CaMKII promotes the occurrence and development of inflammatory responses during myocardial ischemia-reperfusion. Its main mechanism is to activate the nuclear factor-κB (NF-κB) signaling pathway, enhancing the expression of inflammatory factors such as tumor necrosis factor-α (TNF-α) and interleukin-6 (IL-6), thereby aggravating the inflammatory injury of myocardial tissue (Yao et al., 2022). Specifically, the CaMKII-δ9 subtype promotes the phosphorylation of IκBα and activates the NF-κB signaling pathway by directly interacting with the NF-κB inhibitory factor IκBα, leading to a more pronounced inflammatory response (Zhang et al., 2023). In addition, CaMKII regulates the infiltration of inflammatory cells and the polarization state of macrophages, affecting the balance of the myocardial inflammatory microenvironment and further exacerbating myocardial injury induced by ischemia-reperfusion. In ischemia-reperfusion models, CaMKII inhibitors can effectively reduce the expression of inflammatory factors and the infiltration of inflammatory cells (Liu et al., 2019). Therefore, CaMKII mediates inflammatory responses in MIRI by regulating the NF-κB and related inflammatory signaling pathways, making it a potential target for anti-inflammatory therapy.

### 3.3 Involvement in oxidative stress responses

The oxidative activation of CaMKII forms a positive feedback mechanism in MIRI, leading to the massive production of reactive oxygen species (ROS) and promoting oxidative stress responses. ROS are not only a source of CaMKII activation but also, in turn, enhance the continuous activation of CaMKII, creating a vicious cycle that aggravates myocardial cell injury. For example, ox-CaMKII promotes myocardial cell death by affecting the function of ATP-sensitive potassium channels (KATP) (Wu et al., 2019). In addition, mitochondrial dysfunction is a key link in the interaction between ROS and CaMKII. The oxidative activation of CaMKII induces the opening of the mitochondrial membrane permeability transition pore (mPTP), promoting cell apoptosis and necrosis (Wang J. et al., 2021). Intervening in the oxidative state of CaMKII (e.g., using the MMVV mouse model) can reduce MIRI, indicating that blocking this oxidative activation pathway has a protective effect (Wu et al., 2019). Therefore, the CaMKII-mediated oxidative stress response is an important mechanism of MIRI, and therapeutic strategies targeting its oxidative activation are worthy of further research.

### 3.4 Regulation of calcium homeostasisimbalance

CaMKII is a key regulator of intracellular calcium signaling and participates in the regulation of the function of various calcium channels, such as L-type calcium channels and Ryanodine receptors (RyR), thereby affecting the intracellular calcium ion concentration. During myocardial ischemia-reperfusion, the overactivation of CaMKII leads to the abnormal opening of calcium channels, causing calcium overload and inducing myocardial cell contractile dysfunction and cell death (Kong et al., 2019). In addition, CaMKII regulates calcium-handling proteins such as phospholamban (PLN) through phosphorylation, affecting calcium pump activity and calcium ion reuptake to maintain calcium homeostasis (Yao et al., 2022). The abnormal activation of CaMKII also promotes the calmodulin-dependent protein kinase signaling cascade, leading to mitochondrial dysfunction and programmed cell death (Zhang et al., 2023). Calcium homeostasis imbalance is considered a core link in the pathological mechanism of MIRI, and CaMKII plays a key regulatory role in it. Inhibiting CaMKII helps restore calcium homeostasis and reduce ischemia-reperfusion injury (Yu et al., 2019). In summary, CaMKII participates in calcium homeostasis imbalance by regulating calcium channel activity and calcium signaling, promoting myocardial cell dysfunction and necrosis, and is an important target for MIRI.

## 4 Methods

### 4.1 Search strategy

This systematic review was conducted in accordance with the Preferred Reporting Items for Systematic Reviews and Meta-Analyses (PRISMA) standards [60]. The review focused on articles published from January 2000 to September 2025 regarding the beneficial effects of natural metabolites and extracts on myocardial ischemia-reperfusion injury (MIRI) through the regulation of the CaMKII signaling pathway. These articles were identified through dedicated searches of databases including PubMed, Web of Science, CNKI, and Google Scholar. The search keywords encompassed “CaMKII,” “medicinal plants,” “bioactive metabolites,” “natural products,” “phytochemistry,” “traditional Chinese medicine,” “myocardial ischemia-reperfusion injury,” and their combinations.

### 4.2 Inclusion and exclusion criteria for literature

A thorough and systematic literature search was initially carried out across four pivotal databases. This search yielded a total of 254 pertinent records, with 72 originating from PubMed, 77 from Web of Science, 43 from CNKI, and 62 from Google Scholar. The implementation of this multilingual and multi - database search strategy was instrumental in ensuring the comprehensiveness of the initial literature pool. Subsequently, EndNote X9 (Clarivate Analytics) was employed to identify and eliminate duplicate records from the initial set of 254 articles. As a result, 153 unique studies were retained and advanced to the next screening stage. The presence of duplicate entries was primarily attributable to cross - indexing across different databases, and their removal was crucial for guaranteeing both the efficiency of the screening process and the uniqueness of the literature. The subsequent literature screening adhered to three pre - defined stages: title review, abstract evaluation, and full - text assessment. Two researchers independently conducted the title and abstract screening of the 153 de - duplicated studies based on core criteria. Specifically, they assessed whether the research focus was on the association between “natural drugs,” “CaMKII modulation,” and “myocardial ischemia - reperfusion injury (MIRI).” During this stage, 112 articles that were clearly irrelevant were excluded. These included studies with non - myocardial study objects, those lacking CaMKII intervention, or research not centered on natural drugs. Consequently, 41 studies proceeded to the full - text assessment. For the full - text eligibility evaluation, the 41 studies were meticulously scrutinized against strict inclusion criteria. These criteria were as follows: (1) peer - reviewed original English research articles published between January 2000 and October 2025; (2) experimental studies utilizing *in vitro* or animal models; and (3) research on plant - derived extracts or natural metabolites that influence MIRI pathogenesis through CaMKII regulation. Simultaneously, studies that met the exclusion criteria were removed.

These exclusion criteria encompassed review articles, conference abstracts, case reports, editorials, books, posters, studies with ethical or academic integrity issues, and those with incomplete or unpublished data. Ultimately, 25 studies were excluded at this stage for failing to meet the inclusion criteria, such as having unclear mechanisms, lacking CaMKII - related detection, or possessing major flaws in study design. This led to the final inclusion of 16 qualified articles for the review analysis. The entire study selection process, encompassing screening results and reasons for exclusion, was visually represented in Figure 1.

### 4.3 Molecular docking of drugs with CaMKII

The 3D structure of CaMKII was retrieved from the Protein Data Bank (PDB, http://www.pdb.org/) and saved in PDB format. The structures of natural metabolites were obtained from the PubChem database (https://pubchem.ncbi.nlm.nih.gov/) and saved in mol2 format. Water molecules and small-molecule ligands were removed from the protein structure using PyMOL 2.4.1. Subsequently, the processed protein was prepared by adding hydrogen atoms and calculating atomic charges using AutoDock Vina 1.1.2 (Trott and Olson, 2010). Energy minimization of small-molecule metabolites was performed using ChemOffice 22 Professional. For target proteins with known binding ligands, prior to molecular docking, the binding pocket corresponding to the known ligand was extracted as the docking region using the GetBox-PyMOL-Plugin in PyMOL 2.4.1. Finally, molecular docking was carried out using AutoDock Vina 1.1.2, and the binding affinity between CaMKII and each active metabolite was evaluated using docking scores.

## 5 Results

### 5.1 Search results

**FIGURE 3:**
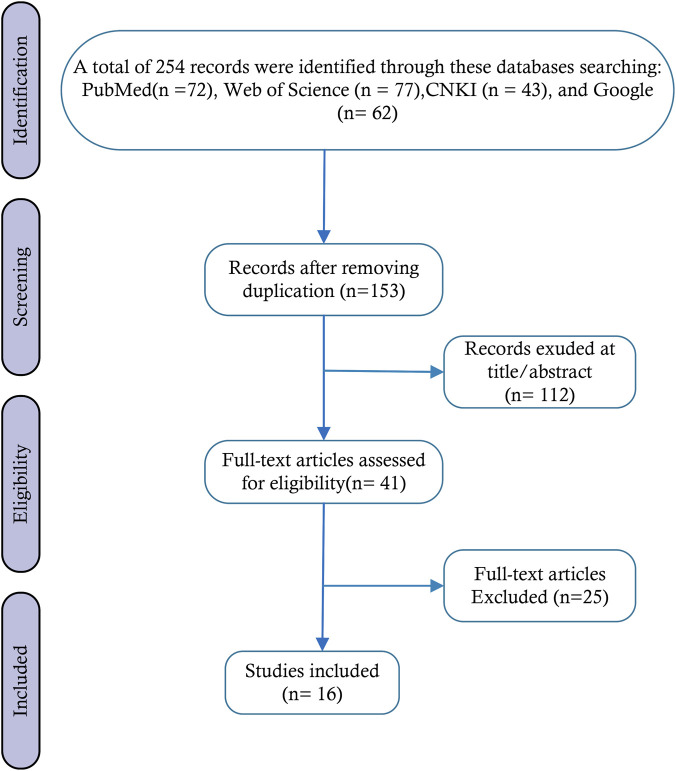
Flow diagram of the study selection process.

**FIGURE 4:**
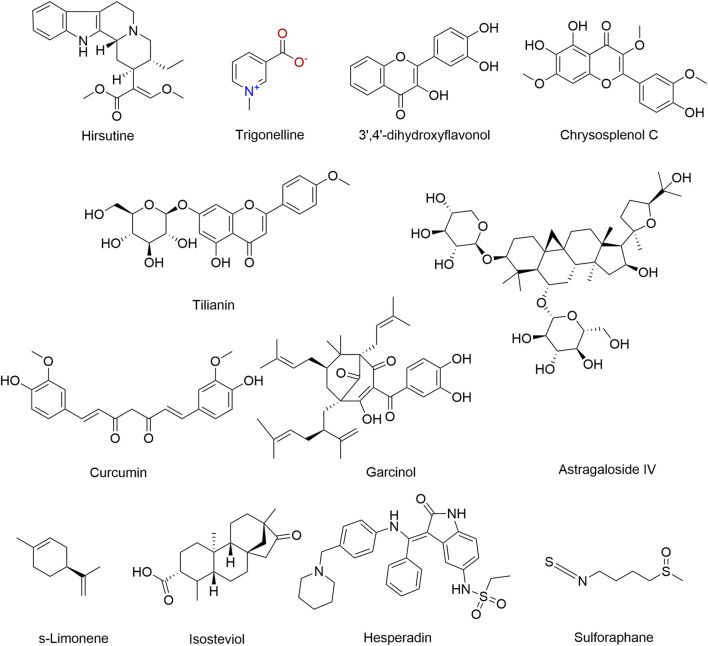
The structural formulae of active ingredients in natural products.

**FIGURE 5:**
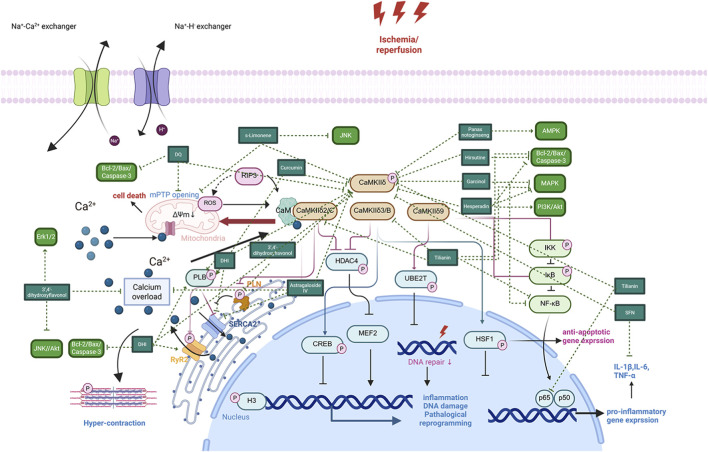
Mechanism of phytochemicals targeting CaMKII for the treatment of MIRI.

Our systematic search identified 254 relevant articles published from January 2000 to September 2025. After a comprehensive review of the reference lists of these studies, 101 duplicate records were removed. Subsequent screening excluded 112 articles that fell outside the scope of our evaluation criteria. Among the remaining 18 studies assessed for eligibility, two were further excluded: (1) studies investigating phytochemical-mediated CaMKII regulation without relevance to MIRI (*n* = 1), and (2) studies on phytochemical neuroprotection in MIRI without exploring CaMKII mechanisms (*n* = 1). No studies examining the CaMKII-MIRI relationship without the involvement of phytochemicals met our inclusion criteria. Additionally, one Chinese article was excluded due to language limitations. This systematic literature review included 16 studies (Figure 3). Comprehensive data collected from the selected studies are presented in Table 1, covering (a) active metabolite classification, (b) natural metabolites, (c) active metabolites, (d) study type, (e) species (gender, weight, n)/cell line, (f) model, (g) intervention, (h) outcomes, (i) effects, and (j) references. The chemical structures of the active metabolites are shown in Figures 4, 5.

**TABLE 1:** The pharmacological activity of natural product targeting CaMKII for the treatment of MIRI.

Active metabolite classification	Natural compounds	Family	Active metabolites	Chemical formula	Study types	Species (Sex. Weight, *n*)/Cell line	Models	Interventions	Results	CAMKII inhibitor	Effects	References
Alkaloid	*Uncaria rhynchophylla* (Miq.) Miq. ex Havil.	Rubiaceae	Hirsutine	C_22_H_28_N_2_O_4_	*In vivo*	Sprague Dawley (SD) rats (male, 280–320 g, NR)	Left Anterior Descending (LAD) ligation	5/10//20 mg/kg/d, [p.o.],15days	↓ p-CaMKII, ↓ p-Drp1, ↑ MFN2, ↓ ROS, ↓ MDA, ↑ SOD, ↓ LDH, ↓ BAX, ↑ BCL-2, ↓ Cleaved Caspase-3, ↓ p-ASK-1, ↓ p-p38 MAPK, ↑ p-AKT, ↑ ATP	NR	Ameliorates myocardial I/R injury, reduces infarct size, and improves cardiac function	Jiang et al. (2023)
Alkaloid	*Trigonella foenum-graecum* L.	Fabaceae	Trigonelline	C_7_H_7_NO_2_	*In vivo*	Wistar rats (NR, 200 ± 10 g, *n* = 7 per group)	ISO-induced	20/40/60 mg/kg/d, [p.o.], 20 days	↓CaMKII δB, ↓CaMKII δC, ↓MDA, ↑SOD, ↑CAT, ↑GPx, ↑GST, ↑GSH, ↓αB-crystallin, ↓Hsp27	NR	Alleviates myocardial injury, reduces oxidative stress, improves cardiac function, and reduces necrosis	Panda et al. (2013)
Flavonoid	*Miliusa balansae*.Finet & Gagnep.	Annonaceae	Chrysosplenol C	C_21_H_20_O_10_	*In vitro*	Ventricular myocytes were isolated from male SD rats (200–300 g)	I/R-induced	50/80 μM, treatment for 2–3 min	↓CaMKII δ, ↑PKC-δ (membrane translocation), ↑SR Ca2+ load, ↑Ca2+ spark frequency	KN-93	Increases cell contraction (exerts a positive inotropic effect)	Wang et al. (2022)
Flavonoid	*Dracocephalum moldavica*.L.	Lamiaceae	Tilianin	C_22_H_22_O_10_	*Ex vivo* and *in vitro*	SD rats (NR,300 ± 20 g, 12); H9c2 cardiomyocytes	I/R injury *via* Langendorff perfusion; OGD/R injury	4 μM; 0.8/4/20/100 μM	↓ox-CaMKII, ↓p-CaMKIIδ, ↓p-JNK, ↓p-p65 (NF-κB), ↓Bax/Bcl-2 ratio, ↑Na+-K+-ATPase activity, ↑ATP concentration, ↓caspase-9 activity, ↓caspase-3 activity	KN-93	Improves cardiac hemodynamic parameters (LVSP, LVDP, ±dp/dtmax/min, heart rate), protects mitochondrial activity, reduces apoptosis, and alleviates inflammation	Jiang H. et al. (2020)
Glycoside	*Astragalus membranaceus* (Fisch.) Bunge	Fabaceae	Astragaloside IV	C_41_H_68_O_14_	*In vivo* and *in vitro*	SD rats (Male, 200–250 g, NR)	Chronic intermittent hypoxia exposure -induced; H/R-induced	40/80 mg/d,4 weeks, [p.o.]; 100 μM	↓p-CaMKII, ↑SERCA2a expression and activity, ↓NCX1, ameliorates Ca^2+^ overload	NR	Improves cardiac function, reduces apoptosis, and alleviates myocardial injury	Jiang S. et al. (2020)
Glycoside	*Panax notoginseng*. (Burkill) F.H.Chen	Araliaceae	Notoginsenoside R1; Ginsenoside Rg1; Ginsenoside Re; Ginsenoside Rb1; Ginsenoside Rd	C_47_H_80_O_18_;C_42_H_72_O_14_;C_48_H_82_O_18_;C_54_H_92_O_23_;C_48_H_82_O_18_	*In vivo* and *in vitro*	C57BL/6 mice (NR, NR); H9c2 cardiomyocytes; HUVECs	LAD ligation; Glucose Deprivation; In vitro platelet aggregation assay	10/100 mg/kg/d, [i.p.],2 weeks; 10/100 μM	↑Autophagy (↑p-AMPK/↑p-CaMKII); ↑Angiogenesis; ↓Platelet aggregation	NR	Improves survival, enhances cardiac function, reduces fibrosis, inhibits cell death, and prevents thrombosis	Wang et al. (2021a)
Polyphenol	*Curcuma longa* L.	Zingiberaceae	Curcumin	C_21_H_20_O_6_	*Ex vivo* and *in vitro*	SD rats (Male, 250–280 g); ARCMs	I/R-induced; H/R-induced	AC3-I@NPs 5 µM, 20 min	↓CaMKII,↓p-PLB	AIP, KN-93	Decreases LDH and CK release in coronary effluents	Liu et al. (2024)
Polyphenol	*Garcinia indica*. (Thouars) Choisy	Clusiaceae	Garcinol	C_38_H_50_O_6_	*In vivo* and *in vitro*	C57Bl/6 mice (NR, NR); AC16 cardiomyocytes	Isoproterenol-induced; Lp(a) stimulation	1 mg/kg/d, [i.p.]; 2.5 µM	↓p- CaMKII, ↓p-ERK, ↓α7-nAChR, ↓p-p38 MAPK, ↓NF-κB, ↓IL-6, ↓TNF-α, ↓CRP, ↓RhoA-GTP, ↑miR-205	​	Alleviates myocardial pathological changes, reduces cardiomyocyte apoptosis, and decreases inflammation levels	Chang et al. (2021)
Terpene	*Citrus* × *limon*. (L.) Osbeck	Rutaceae	s-Limonene	C_10_H_16_	*In vivo*	Wistar rats (200–250 g); isolated rat cardiomyocytes	ISO-induced	1 mg/kg/d, [ i.p]; 1 mg/kg, 30 min	↓ox-CaMKII, ↑SOD activity, ↓Cytoplasmic ROS, ↓mitochondrial ROS, ↑GPx	NAC	Attenuates myocardial injury, reduces infarct size and collagen content, prevents ST-segment elevation and QTc prolongation, and reduces arrhythmias	Rhana et al. (2022)
Other	*Citrus* × *sinensis*. (L.) Osbeck	Rutaceae	Hesperadin	C_21_H_20_FN_3_O_3_	*In vivo* and *in vitro*	C57BL/6 mice (NR, NR) and BALB/c nude mice (NR, NR); NRCMs	Permanent LAD ligation; Ischemia/Reperfusion	2.5 μg/kg/d, [i.p.], 4 weeks; 0.2 and 0.5 μmol/L, 48 h	↓ p-CaMKII-δ,↓ CaMKII-δ9, ↓cleaved caspase-3, ↓γH2AX	KN-93	Improves cardiac function, reduces cardiac hypertrophy, decreases cell death, and reduces DNA damage	Zhang et al. (2022b)
Other	*Brassica oleracea*.L.	Brassicaceae	Sulforaphane	C_6_H_11_NOS_2_	*In vivo* and *in vitro*	C57BL/6 mice (NR, NR) and Balb/c mice (*n* = 6); H9c2 cardiomyocytes	LAD ligation/reperfusion; H/R-induced	50 μg/kg/d, [i.p.], 3 days; 5 μM pretreatment for 3 h	↑CaMKIIN2, ↓p-CaMKIIδ, ↓IL-1β, ↓IL-6, ↓TNF-α, ↑Nrf2	NR	Reduces myocardial infarct size, improves cardiac function (increases EF and FS), and alleviates myocardial ultrastructural damage	Zhang et al. (2022a)
Other	Not applicable	Not applicable	3′,4′-dihydroxyflavonol	C_15_H_10_O_5_	*In vivo* and *in vitro*	Sheep; C2C12 cells; Primary cardiomyocytes	I/R-induced; H_2_O_2_ -induced	6.6 mg/kg/d [i.v.]; 10 μM pre-treatment	↓ CaMKIIδ, ↓p-p38 MAPK, ↓p-JNK, ↓p-MKK3/6, ↓p-MKK4/7	KN-93	Reduces myocardial infarct size and increases cell viability	Lim et al. (2013)
Other	Not applicable	Not applicable	3′,4′-dihydroxyflavonol	C_15_H_10_O_5_	*Ex vivo* and *in vitro*	SD rats (male,250–300 g,NR); H9C2 cardiomyocytes	I/R-induced	10 μM	↓ CaMKIIδ, ↓p-PLN, ↓p-JNK2, ↑p-Erk1/2, ↓p-Akt	KN- 93	Improves cardiac function, reduces LDH release, and reduces apoptosis	Chin et al. (2018)
Traditional Chinese medicine	Not applicable	Not applicable	Chinese medicine compound (containing Salvia miltiorrhiza, Carthamus tinctorius, etc.)	Not applicable	*In vivo*	SD rats (male, 200–250 g, 54)	LAD ligation/Hypoxia	0.42/1.05/2.1 mL/kg/d, [i.p.], 4 weeks	↓CaMKII, ↑p-PLB, ↑SERCA2a activity, ↓Bax, ↓cleaved caspase-3	NR	Reduces infarct size, improves cardiac function, relieves calcium overload, and inhibits apoptosis	Zhang et al. (2021b)
Traditional Chinese medicine	Not applicable	Not applicable	FMAI (protocatechuic acid, cryptotanshinone, borneol, eugenol)	Not applicable	*In vivo* and *in vitro*	SD rats (Male, 280–300 g,60); Neonatal rat cardiomyocytes	LAD ligation/Hypoxia; H/R injury -induced	3.25/6.5/13 mg/kg/d, [p.o.],4 weeks; 3.25/6.5/13 μg/mL	↓CaM, ↓CaMKIIδ, ↑RyR2, ↑PLB	NR	Inhibits apoptosis, reduces infarct size, decreases serum CK, LDH, and AST levels, and increases SOD activity	Liu et al. (2017)
Traditional Chinese medicine	Not applicable	Not applicable	Salvianolic acids, Panax notoginseng saponins	Not applicable	*In vivo* and *in* *vitro*	SD rats (male, 280–300 g, NR); H9c2 cardiomyocytes	LAD ligation/Hypoxia; H_2_O_2_-induced oxidative stress injury	0.6/1.2 g/kg/d, [ i.g.], 5 days; 150/300/600 mg/mL, pretreatment for 2.5 h.	↓p-CaMKII, ↓p-Drp-1, ↓ROS, ↑GSH, ↑GPx, ↓MDA, ↓Bax/Bcl-2 ratio, ↓cleaved-Caspase-3;↓ROS, ↓mPTP opening, preserved mitochond*rial membrane potential, ↓Cytochrome c release, ↓Caspase-3/8/9	NR	Reduces myocardial infarct size, decreases serum cTnT and LDH levels, increases cardiomyocyte viability, and reduces apoptosis; increases cell viability, reduces apoptosis, and alleviates oxidative stress	Yang et al. (2025)

## 6 Targeting CaMKII: natural metabolites for the potential treatment of MIRI

Myocardial ischemia-reperfusion injury (MIRI) remains a critical challenge in cardiovascular therapy, characterized by exacerbated tissue damage following the restoration of blood flow to ischemic myocardium. Cumulative evidence highlights the aberrant activation of CaMKII as a key mediator of MIRI, driving pathways such as oxidative stress, calcium overload, mitochondrial dysfunction, and cardiomyocyte apoptosis. In recent years, natural metabolites have emerged as attractive candidates for MIRI treatment, owing to their multi-targeted bioactivities, favorable safety profiles, and potential to modulate CaMKII signaling cascades. This review systematically summarizes the therapeutic potential of natural metabolites from diverse chemical classes—including alkaloids, flavonoids, glycosides, polyphenols, terpenes, and traditional Chinese medicine (TCM) formulations—in targeting CaMKII to alleviate MIRI.

### 6.1 Alkaloids

#### 6.1.1 Hirsutine

*Uncaria rhynchophylla (Miq.) Miq. ex Havil.*, a well-documented traditional Chinese medicinal botanical drug, has long been utilized in the management of cardiovascular disorders. Clinical observations indicate that its extracts exhibit 83% efficacy in patients with mild-to-moderate hypertension and confer protection against MIRI by reducing infarct size and mitigating oxidative stress. Hirsutine (C_22_H_28_N_2_O_4_; molecular weight [MW] 384.47) (Bhuia et al., 2023)., a principal bioactive alkaloid isolated from *U. rhynchophylla*, possesses robust antioxidant, anti-apoptotic, and cardioprotective properties (Wu et al., 2011).

In a rat model of myocardial I/R injury, hirsutine was administered *via* gavage to sham-operated, I/R-injured, and low- (5 mg/kg), medium- (10 mg/kg), and high-dose (20 mg/kg) pretreatment groups for 15 days prior to I/R induction. Dose-dependent reductions in infarct size were observed, with the high-dose group demonstrating marked improvements in myocardial structural integrity, left ventricular ejection fraction (LVEF), and left ventricular fractional shortening (LVFS), alongside decreased left ventricular end-systolic diameter (LVESD) and left ventricular end-diastolic diameter (LVEDD). Biochemical analyses revealed that hirsutine dose-dependently lowered serum lactate dehydrogenase (LDH) and malondialdehyde (MDA) levels, attenuated myocardial reactive oxygen species (ROS) and succinate accumulation, enhanced superoxide dismutase (SOD) activity and adenosine triphosphate (ATP) content, and restored the activity of mitochondrial complexes I–IV. Additionally, hirsutine suppressed cardiomyocyte apoptosis, as evidenced by reduced numbers of TUNEL-positive cells in the high-dose group.

Mechanistically, hirsutine downregulates I/R-induced CaMKII phosphorylation, modulates mitochondrial dynamics by upregulating mitofusin 2 (Mfn2) and reducing dynamin-related protein 1 (Drp1) phosphorylation, and inhibits the AKT/ASK-1/p38 MAPK signaling pathway. These effects collectively downregulate pro-apoptotic proteins (BAX, active caspase-3) and upregulate the anti-apoptotic protein BCL-2 (Jiang et al., 2023). Notably, hirsutine improves mitochondrial function by regulating upstream calcium signaling rather than directly interacting with CaMKII activation sites, underscoring its potential as a novel natural product candidate for MIRI treatment *via* CaMKII modulation.

#### 6.1.2 Trigonelline

Trigonelline (TRG; C_7_H_7_NO_2_; MW 137.14), an alkaloid derived from *Trigonella foenum-graecum* L. seeds, exhibits diverse biological activities including antibacterial, hypoglycemic, and anticancer effects (Khalili et al., 2018). Its favorable water solubility facilitates purification, and emerging evidence indicates potent antioxidant, anti-inflammatory, and cardioprotective properties, prompting investigations into its efficacy in isoproterenol (ISO)-induced myocardial injury.

In a rat model of ISO-induced myocardial injury, administration of TRG at 40 mg/kg optimally reversed ST-segment elevation, tachycardia, and elevated serum cardiac biomarkers (creatine kinase-MB [CK-MB], LDH, cardiac troponin T [cTnT]). Remarkably, TRG reduced myocardial infarct size from 70.2% (ISO group) to 23.2%, restored the activity of antioxidant enzymes (SOD, catalase [CAT], glutathione peroxidase [GPx]), and attenuated lipid peroxidation (MDA). Histopathological analyses confirmed reduced myocardial fiber damage and inflammatory infiltration. Proteomic studies further linked TRG’s cardioprotective effects to the downregulation of heat shock protein 27 (Hsp27), αB-crystallin, and CaMKII δB/δC isoforms (Panda et al., 2013).

Mechanistically, TRG mitigates ISO-induced oxidative stress and cardiomyocyte injury by inhibiting the aberrant activation of CaMKII δB/δC isoforms and suppressing the overexpression of Hsp27/αB-crystallin. Its protective effects are likely mediated through modulation of calcineurin (CnA)-dependent signaling pathways and maintenance of intracellular calcium homeostasis. These findings establish TRG as a promising natural candidate for myocardial infarction therapy, leveraging synergistic antioxidant and anti-inflammatory activities *via* targeted inhibition of CaMKII δB/δC.

### 6.2 Flavonoids

#### 6.2.1 Chrysosplenol C

Chrysosplenol C (C_17_H_16_O_8_; MW 348.30), a natural flavonoid isolated from *Miliusa balansae Finet & Gagnep.* —the latter traditionally used to treat gastric and glomerular nephropathies—exhibits positive inotropic effects, enhancing myocardial contractility and holding promise for cardiovascular applications (Son et al., 2011). It is highly soluble in DMSO, with stock solutions typically diluted in Tyrode’s solution for cellular experiments.

Pharmacological studies using rat ventricular myocytes (extracellular perfusion) investigated the effects of Chrysosplenol C (1–200 μM) alone or in combination with kinase inhibitors (PKC inhibitors: chelerythrine, GF109203X; CaMKII inhibitors: KN-93, inactive analog KN-92). Chrysosplenol C dose-dependently increased Ca^2+^ transient amplitude (half-maximal effective concentration [EC_50_] ≈ 21 μM), peaking at 58% with 80 μM treatment, while elevating resting Ca^2+^ spark frequency/area and sarcoplasmic reticulum (SR) Ca^2+^ content without altering L-type Ca^2+^ current or transient decay rates. PKC inhibitors abolished these effects, whereas KN-93 partially attenuated them, and KN-92 had no impact (Wang et al., 2022).

Western blot analyses revealed rapid translocation of PKC-δ to membrane fractions (peaking at 2 min) and delayed activation of PKC-α, with no effect on PKC-ε. CaMKII activity remained unchanged, suggesting its partial involvement is dependent on upstream PKC activation. Mechanistically, Chrysosplenol C enhances SR Ca^2+^ loading and release *via* PKC (primarily the δ-subtype), thereby promoting cardiomyocyte contractility. This PKC-dependent, CaMKII-modulated pathway distinguishes it from direct CaMKII inhibitors such as KN-93, offering a novel therapeutic strategy for myocardial dysfunction.

#### 6.2.2 Tilianin

Tilianin (C_22_H_26_O_10_; MW 466.44), a natural flavonoid glycoside isolated from *Dracocephalum moldavica L.* and Agastache rugosa—plants used in traditional Chinese medicine and health beverages (Wang et al., 2018)—exhibits cardiovascular protective effects, including anti-inflammatory, anti-hypoxic, anti-apoptotic, and mitochondrial protective activities (Akanda et al., 2019; Hameed Ali et al., 2023).

*In vitro* studies using H9c2 cardiomyocytes subjected to oxygen-glucose deprivation/reoxygenation (OGD/R) and *ex vivo* experiments with rat hearts exposed to I/R injury were conducted. Tilianin was administered *via* cell pre-incubation (0.8–100 μM) or heart perfusion (4 μM), with KN-93 (5 μM for cells, 2.5 μM for hearts) as a positive control. Results showed that tilianin improved H9c2 cell viability, reduced LDH release, and regulated the expression of apoptosis- and inflammation-related molecules. In *ex vivo* hearts, tilianin enhanced cardiac function, increased Na^+^-K^+^-ATPase activity and ATP content, and reversed aberrant expression of signaling molecules. Notably, these protective effects were abrogated by KN-93, confirming CaMKII as a direct molecular target.

Molecular docking and kinase assays revealed that tilianin binds non-competitively to CaMKIIδ, reducing its phosphorylation and oxidation. By inhibiting CaMKIIδ, tilianin blocks the JNK/NF-κB and mitochondrial apoptotic pathways. Although tilianin exhibits weaker *in vitro* inhibitory activity against CaMKII compared to KN-93, it provides more comprehensive cardioprotection. These findings demonstrate that tilianin alleviates MIRI by targeting CaMKIIδ, offering a safe and effective candidate for myocardial infarction treatment that overcomes the limitations of traditional CaMKII inhibitors (Jiang H. et al., 2020).

### 6.3 Glycosides

#### 6.3.1 Astragaloside IV

Astragaloside IV (AS-IV; C_44_H_72_O_14_; MW 808.04), the major bioactive metabolite of *Astragalus membranaceus (Fisch.) Bunge* (Zhang et al., 2006)—a traditional Chinese medicine widely used for cardiovascular disorders—possesses antioxidant, anti-inflammatory, and anti-apoptotic properties, with demonstrated protection against myocardial ischemia and hypertrophy (Yang et al., 2022). It is soluble in water, methanol, and ethanol, and its core biological effects include the regulation of calcium homeostasis, inhibition of apoptosis, and attenuation of myocardial fibrosis (Zhang et al., 2020).

In a rat model and complementary H9C2 cell experiments, SD rats exhibited left ventricular dysfunction, myocardial structural disorganization, fibrosis, apoptosis, Ca^2+^ overload, and aberrant expression of calcium-handling proteins (SERCA2a, RYR2) and signaling molecules (phosphorylated CaMKII [p-CaMKII], NCX1). Administration of high-dose AS-IV (80 mg/kg) significantly improved cardiac function, reduced histological damage and apoptosis, and reversed abnormalities in Ca^2+^ homeostasis and protein expression compared to low-dose AS-IV (40 mg/kg). *In vitro*, pre-treatment of H9C2 cells with 100 μM AS-IV for 48 h inhibited cell apoptosis, reduced Ca^2+^ accumulation, and normalized the expression of key regulatory proteins (Jiang S. et al., 2020).

Mechanistically, AS-IV modulates upstream calcium signaling to alleviate Ca^2+^ overload, inhibits aberrant CaMKII phosphorylation, restores the function of SERCA2a and RYR2, and downregulates NCX1 expression—collectively suppressing cardiomyocyte apoptosis and myocardial fibrosis. These findings support AS-IV as a potential therapeutic candidate for obstructive sleep apnea syndrome (OSAS)-related myocardial injury and myocardial infarction. However, further studies are required to clarify its specificity for CaMKII isoforms and comparative efficacy with classical CaMKII inhibitors.

#### 6.3.2 Panax notoginseng saponins

Panax notoginseng saponins (PNS), the principal bioactive metabolites extracted from the roots of *Panax notoginseng (Burkill) F.H.Chen*, have been widely employed in traditional Chinese medicine for the treatment of cardiovascular-related disorders (Uzayisenga et al., 2014). Pharmacologically, PNS exhibits a diverse range of effects, including the inhibition of platelet aggregation, the promotion of endothelial cell migration, and the stimulation of angiogenesis. Collectively, these effects confer its cardioprotective properties (Jin et al., 2019; Wang et al., 2021a).

In a murine model of AMI, PNS treatment significantly improved survival rates, enhanced cardiac function, reduced the area of fibrotic scarring, increased left ventricular wall thickness, and decreased cardiomyocyte size. *In vitro* experiments using H9c2 cardiomyocytes subjected to glucose deprivation (GD) stress confirmed that PNS and its metabolites (notably Rg1) enhanced autophagic flux, a critical pro-survival mechanism. Mechanistic studies revealed that this protective effect was mediated through the phosphorylation of key signaling molecules—specifically, CaMKII at threonine 287 (Thr287) and AMP-activated protein kinase (AMPK) at threonine 172 (Thr172). Consequently, PNS intervention shifted cellular responses toward adaptive autophagy, thereby reducing cell death. Compared with the model control group, the PNS-treated group exhibited significantly improved phenotypic outcomes, which correlated with the upregulation of pathways associated with autophagy and angiogenesis (Wang et al., 2021a).

### 6.4 Polyphenols

#### 6.4.1 Curcumin

Curcumin is a natural polyphenolic metabolite extracted from the rhizome of *Curcuma longa L.*, serving as the primary bioactive metabolite responsible for its pharmacological effects (Valizadeh Kiamahalleh et al., 2016; Umapathy et al., 2022). Widely used in traditional medicine for anti-inflammatory and antioxidant therapies, curcumin has the molecular formula C_21_H_20_O_6_ and a molecular weight of 368.38 (Menon and Sudheer, 2007). As a hydrophobic metabolite, it exhibits poor water solubility. Curcumin demonstrates a broad spectrum of pharmacological activities, including notable anti-inflammatory, antioxidant, anti-apoptotic, and cardioprotective properties (Kuzminska et al., 2024).

Recent studies have explored its synergistic potential with AC3-I—a synthetic CaMKII-inhibitory peptide derived from the CaMKII autoinhibitory domain—in the development of a nanodelivery system (AC3-I@HSA-CCM NPs) for targeted cardioprotection. This hybrid formulation enhances intracellular delivery and co-targets CaMKII hyperactivation and oxidative stress.

In rat cardiomyocyte H/R models, AC3-I@HSA-CCM NPs reduced cell death, LDH release, and p-PLB levels more effectively than curcumin alone. *Ex vivo* experiments demonstrated that the nanosystem lowered I/R-induced LDH and CK levels, matching the efficacy of the classic CaMKII inhibitor AIP while exhibiting superior specificity compared to KN-93. Mechanistically, AC3-I directly blocks the substrate-binding site of CaMKII to restore calcium homeostasis, while curcumin mitigates oxidative stress. AC3-I (MW ∼1,526.87 Da) requires nanoformulation due to membrane impermeability, and covalent conjugation with HSA-CCM NPs enables co-delivery to cardiomyocytes (Liu et al., 2024).

This “synthetic + natural” strategy bridges artificial and natural pharmacology, offering a scalable, dual-targeted approach for CaMKII-mediated cardioprotection in MIRI. Future directions focus on optimizing the biodistribution of AC3-I@HSA-CCM NPs and advancing clinical trials to validate their efficacy in human myocardial infarction.

#### 6.4.2 Garcinol

Garcinol (C_38_H_54_O_6_; MW 606.83), a natural polyphenol isolated from the fruits and bark of *Garcinia indica (Thouars) Choisy* (Desai et al., 2022), has a traditional history of use for anti-inflammatory and antibacterial purposes. It exhibits antioxidant, anti-inflammatory, anti-apoptotic, and anti-tumor activities, with emerging potential for the treatment of AMI *via* modulation of CaMKII signaling (Meng et al., 2013).

In cell experiments using Lp(a)-stimulated AC16 cardiomyocytes, garcinol (0.5–2.5 μM) dose-dependently reversed Lp(a)-induced (1–10 μM) reductions in cell viability, increases in apoptosis rate, accumulation of ROS and mitochondrial superoxide (mtO_2_^-^), and upregulation of inflammatory factors (IL-6, TNF-α, CRP, NF-κB) and kinase phosphorylation (CaMKII, ERK, p38 MAPK). These effects were comparable to those of the p38 MAPK inhibitor SB203580. In a mouse model of ISO-induced AMI, i.p. administration of garcinol (1 mg/kg) reduced heart and liver weights, alleviated myocardial pathological damage, decreased apoptotic cell numbers, and lowered levels of inflammatory factors and kinase phosphorylation compared to the Lp(a)-treated group (1.5 mg/kg), while upregulating miR-205 (Chang et al., 2021).

Mechanistically, garcinol indirectly inhibits CaMKII phosphorylation *via* the α7-nicotinic acetylcholine receptor (α7-nAChR)-mediated pathway, downregulating downstream inflammatory and ERK signaling. It also suppresses the RhoA-GTP/ROCK and IGF2R pathways to reduce oxidative stress and apoptosis. Notably, garcinol regulates CaMKII by upregulating miR-205, which inhibits α7-nAChR expression, rather than directly interacting with CaMKII activation sites. These findings establish garcinol as a natural candidate for AMI treatment, exerting protective effects against Lp(a)-induced myocardial damage *via* the α7-nAChR/miR-205/CaMKII axis.

### 6.5 Terpenes

#### 6.5.1 s-Limonene

s-Limonene (SL; C_10_H_16_; MW 136.23), a widely distributed monoterpene found in the essential oils of citrus fruit peels and other natural plants, has a long history of application in food, cosmetics, and traditional botanical drugal medicine (Wróblewska, 2014). Emerging evidence indicates its potential for cardiovascular protection, with its isomer D-limonene demonstrating anti-apoptotic effects in an ISO-induced MI model—providing a foundation for investigations into SL’s cardioprotective efficacy (Bedjanian et al., 2014).

In an ISO-induced rat MI model, i.p. administration of SL (1 mg/kg) improved key phenotypic parameters, including reduced infarct size, lowered total CK and CK-MB levels, ameliorated ECG abnormalities, alleviated myocardial tissue damage, restored left ventricular developed pressure (LVDP), reduced arrhythmia scores, and inhibited Ca^2+^ overload and ROS generation. At the molecular level, SL restored SOD and GPx activities and reduced oxiCaMKII expression. Compared to N-acetylcysteine (NAC; 100 mg/kg i.p.), SL exhibited comparable efficacy at a much lower dose and demonstrated superior safety (LD_50_ ∼3.6 g/kg i.p. in rodents) (Rhana et al., 2022).

Mechanistically, SL regulates upstream calcium signaling by inhibiting Ca^2+^ overload, rather than directly binding to CaMKII sites. Its cardioprotective effects are mediated through synergistic antioxidant, anti-inflammatory, and calcium homeostasis-regulating activities. These findings position SL as a potential adjuvant therapeutic agent for MI, leveraging its ability to reduce Ca^2+^ overload, suppress ROS generation, restore antioxidant enzyme function, and lower CaMKII oxidation.

### 6.6 Other metabolites

#### 6.6.1 Hesperadin

Hesperadin is a characteristic metabolite found in citrus fruits (family *Rutaceae*), such as oranges (*Citrus sinensis*), grapefruits (*Citrus paradisi*), mandarins (*Citrus reticulata*), limes (*Citrus aurantifolia*), and lemons (*Citrus limon*) (Sessa et al., 2005). With the molecular formula C_30_H_32_N_4_O_3_S and a molecular weight of 528.67 g/mol, it exhibits good lipophilicity (Shamsipour et al., 2014). This metabolite demonstrates significant cardioprotective properties by attenuating myocardial injury through anti-apoptotic mechanisms.

Hesperadin exhibits cardioprotective effects *via* anti-apoptotic, DNA damage-inhibitory, and CaMKII-regulatory mechanisms. In animal models, administration of hesperadin (2.5 μg kg^−1^·d^−1^ i.p.) and *in vitro* treatment (0.2–0.5 μmol/L) reduced CaMKII-δ9 overexpression- and H/R-induced apoptosis in neonatal rat ventricular myocytes (NRVMs) and human stem cell-derived cardiomyocytes, as evidenced by decreased caspase 3/7 activity, LDH release, and cleaved caspase 3 levels. It also suppressed DNA damage (γH2AX) and CaMKII-δ phosphorylation (IC_50_ = 0.21 μmol/L). In murine I/R models, both pretreatment and posttreatment with hesperadin reduced infarct size, lowered serum LDH levels, decreased the number of TUNEL/γH2AX-positive cells, improved EF/FS, and alleviated myocardial remodeling.

Hesperadin selectively targets CaMKII-δ (particularly the δ9 isoform), with 17–200-fold higher potency against CaMKII-δ compared to CaMKII-α/β/γ (IC_50_ = 0.073 μmol/L vs. 1.259–16.218 μmol/L). It inhibits CaMKII-δ splice variants (δ2/δ3/δ9; IC_50_ = 32–43 nmol/L) by binding to the ATP-competitive site (binding energy = −8.82 kcal/mol), suppressing activation independently of calcium signaling. Unlike KN-93, which non-specifically blocks ion channels and indirectly inhibits CaMKII *via* Ca^2+^/CaM competition, hesperadin exhibits no off-target effects and achieves superior CaMKII-δ inhibition (cell-free IC_50_ = 65 nmol/L vs. KN-93’s 20.9 μmol/L; inhibition efficiency 96.21% vs. 63.74%). The absence of cardiotoxicity at therapeutic doses further supports hesperadin as a promising candidate for ischemic heart disease (Zhang et al., 2022b).

#### 6.6.2 Sulforaphane

Sulforaphane (SFN) is an isothiocyanate-class bioactive metabolite widely present in cruciferous vegetables, such as broccoli and Chinese kale (*Brassica oleracea L.*) (Fimognari et al., 2008). With the molecular formula C_6_H_11_NOS_2_ and a molecular weight of 177.3, SFN exhibits significant antioxidant, anti-inflammatory, and anti-apoptotic properties. Initially studied extensively for its anticancer effects, SFN has recently demonstrated potential in cardiovascular protection (Baralić et al., 2024; Hajimohammadi et al., 2024).

In experimental models, SFN was administered at 50 mg/kg (i.p.) in animals and 5 μmol/L in cells. In H9c2 cardiomyocyte H/R models, SFN enhanced cell viability (CCK-8 assay), reduced pro-inflammatory cytokine levels, preserved mitochondrial membrane potential, and inhibited Ca^2+^ overload. Mechanistically, SFN upregulated CaMKIIN2 (an endogenous CaMKIIδ inhibitor) and downregulated total and phosphorylated CaMKIIδ (T287). CaMKIIN2 knockdown abolished SFN’s protective effects, while CaMKIIN2 overexpression mimicked them. In mouse I/R models, SFN improved EF/FS, reduced left ventricular internal dimension at systole (LVIDS) and infarct size (HE staining), and alleviated mitochondrial and myofibril damage (transmission electron microscopy [TEM]). It also decreased serum cytokine levels and CaMKIIδ/p-CaMKIIδ expression, while upregulating CaMKIIN2/Nrf2 to near-normal levels compared to I/R controls (Zhang et al., 2022a).

SFN selectively targets cardiac CaMKIIδ, with no reported effects on CaMKIIα/β/γ, indicating subtype specificity. Unlike direct CaMKII inhibitors, SFN indirectly suppresses CaMKIIδ *via* CaMKIIN2 upregulation rather than targeting the catalytic site. It also mitigates H/R-induced Ca^2+^ overload, reducing CaMKIIδ hyperactivation. By integrating CaMKIIN2/CaMKIIδ modulation, calcium homeostasis regulation, and anti-inflammatory/antioxidant effects, SFN attenuates MIRI, positioning it as a natural candidate for CaMKII-targeted ischemic heart therapy.

#### 6.6.3 3′,4′-dihydroxyflavonol

3′,4′-Dihydroxyflavonol (DiOHF), a synthetic flavonol analogue characterized by 3′,4′-dihydroxy substituents, exhibits potent antioxidant and kinase-modulating activities. Its prodrug formulation, NP202, enables intravenous administration in large animal models, while DiOHF itself is soluble in dimethyl sulfoxide (DMSO) for *in vitro* studies. Structural analyses emphasize the critical role of the 3′,4′-dihydroxy moieties in mediating its bioactivity (Lim et al., 2013).

In ovine myocardial I/R models, NP202 significantly reduced infarct size and inhibited JNK/p38 MAPK phosphorylation without affecting protective ERK/Akt signaling cascades. *In vitro* experiments demonstrated that DiOHF protects cardiomyocytes from oxidative stress by suppressing MKK4/7 and MKK3/6. Mass spectrometry and enzymatic assays confirmed direct binding of DiOHF to CaMKIIδ (half-maximal inhibitory concentration [IC_50_] = 0.25 μM), outperforming the classical CaMKII inhibitor KN-93 (IC_50_ = 3.3 μM). DiOHF inhibits CaMKIIδ through dual mechanisms: competition for the ATP-binding site and blockade of Ca^2+^/calmodulin (CaM) binding (Lim et al., 2013). These actions reduce phospholamban (PLN)-mediated Ca^2+^ leakage and JNK2-driven apoptosis. In rat hearts, DiOHF improved cardiac contractility and suppressed the activation of CaMKIIδ/PLN/JNK2 signaling (Chin et al., 2018).

Collectively, DiOHF exerts cardioprotective effects in MIRI by directly targeting and inhibiting CaMKIIδ, thereby modulating downstream MAPK signaling, regulating PLN-mediated calcium homeostasis, and suppressing JNK2-driven apoptosis. Its superior therapeutic efficacy compared to KN-93, combined with its lack of interference with protective kinase cascades, positions DiOHF as a highly effective natural product-derived agent with a well-defined molecular target for myocardial infarction treatment.

### 6.7 Traditional Chinese medicine formulations

#### 6.7.1 Danhong injection (DHI)

Danhong Injection is a traditional Chinese medicine (TCM) metabolite formulation widely used in clinical treatment of cardiovascular diseases (Guo et al., 2020; Chen et al., 2022). Composed primarily of *Salvia miltiorrhiza* (Danshen) and *Carthamus tinctorius* (Honghua), it exerts effects of promoting blood circulation, removing blood stasis, dredging meridians, and relaxing collaterals, demonstrating promising therapeutic potential in myocardial infarction management (Zheng et al., 2025).

In a rat MI model, DHI was administered *via* i.p. injection at low, medium, and high doses, with trimetazidine as a positive control. All doses of DHI reduced infarct size (with a dose-dependent effect in the high-dose group), improved cardiac function, and lowered serum cardiac injury markers. Histopathological analyses revealed reduced numbers of apoptotic cardiomyocytes, downregulated expression of Bax, cleaved caspase-3, and total CaMKII protein. High-dose DHI exhibited comparable efficacy to TMZ. At the cellular level, DHI improved cardiomyocyte shortening, Ca^2+^ transients, and SR Ca^2+^ content, while upregulating the expression of calcium-handling proteins (Zhang et al., 2021b).

Although the study only detected total CaMKII protein, the dominance of CaMKIIδ in myocardial tissue suggests that DHI likely targets this isoform. Mechanistically, DHI indirectly inhibits CaMKII by downregulating its total protein expression and activates PKA to promote PLB phosphorylation, enhancing SERCA2a-mediated Ca^2+^ uptake and improving calcium homeostasis—thereby reducing Ca^2+^ overload-induced myocardial damage. Collectively, DHI inhibits apoptosis *via* CaMKII downregulation and improves calcium handling and contractile function *via* the PKA-PLB-SERCA2a pathway, exerting anti-myocardial infarction effects through multiple synergistic mechanisms. These findings provide support for the modernization of TCM metabolites and their clinical application in MIRI treatment.

#### 6.7.2 Guanxin Shutong capsule

Guanxin Shutong Capsule is a TCM metabolite widely used in the treatment of myocardial ischemia (Wang et al., 2021b). Its core active metabolites include protocatechuic acid, cryptotanshinone, borneol, and eugenol (abbreviated as FMAI). This formulation exerts cardioprotective effects through multiple mechanisms, encompassing anti-inflammatory, antioxidant stress, and anti-apoptotic pharmacological activities (Liu et al., 2017).

In experimental studies, FMAI was administered *via* different routes: cellular incubation (specific concentrations and dose ratios) and gavage (varying doses) in animal models, with simvastatin as a positive control. At the cellular level, in a neonatal rat ventricular myocyte H/R model, FMAI increased cell viability, reduced apoptosis, and inhibited Ca^2+^ overload. It also downregulated the expression of CaM and CaMKIIδ, while upregulating RyR2 and PLB—with superior effects compared to single-metabolite groups. At the animal level, in a Sprague-Dawley rat MI model, high-dose FMAI reduced infarct size, lowered serum cardiac markers, and increased SOD activity, whereas low and medium doses were ineffective. Simvastatin exhibited stronger efficacy in some parameters (Liu et al., 2017).

FMAI specifically targets CaMKIIδ in myocardial tissue. Mechanistically, it indirectly downregulates CaMKIIδ by inhibiting upstream CaM, reduces Ca^2+^ overload, and upregulates RyR2 and PLB to maintain calcium homeostasis. These findings demonstrate that FMAI exerts anti-myocardial infarction effects through multiple synergistic pathways, providing a basis for the research and clinical application of TCM metabolites targeting CaMKII.

#### 6.7.3 Danqi soft capsule

Danqi Soft Capsule (DQ) is a classical TCM metabolite composed of *Salvia miltiorrhiza Bunge* (Danshen) and *Panax notoginseng (Burk.) F. H. Chen* (Sanqi) (Ma et al., 2019). It is widely used in the clinical treatment of cardiovascular and cerebrovascular diseases, demonstrating significant potential in alleviating myocardial I/R injury. Its pharmacological effects primarily include antioxidant, anti-apoptotic, and anti-inflammatory properties, exerting protective effects in animal and cellular models through oral administration (Zhang et al., 2016; Ghasemi-Dehnoo et al., 2022).

In experimental models, DQ was administered *via* gavage at doses of 0.6 and 1.2 g/kg (animals) or used at concentrations of 300 and 600 μg/mL (cells). In animal MI models, DQ reduced infarct size, lowered serum cardiac markers (cTnT, LDH), alleviated pathological damage, decreased apoptosis, regulated the expression of apoptosis-related proteins, maintained mitochondrial structure, and downregulated the expression of p-CaMKII and p-Drp-1. In cell models, DQ improved cell viability, reduced apoptosis and ROS generation, preserved mitochondrial membrane potential, enhanced mitochondrial respiratory function, and regulated the expression of key signaling proteins. High-dose DQ exhibited superior efficacy compared to low-dose in all parameters (Yang et al., 2025).

Mechanistically, DQ regulates upstream calcium signaling to reduce CaMKII phosphorylation and inhibits Drp-1 phosphorylation *via* downregulation of p-CaMKII, thereby maintaining mitochondrial homeostasis. Further studies are required to compare its efficacy with classical CaMKII inhibitors. Overall, DQ exerts anti-MIRI effects through multiple synergistic pathways, providing a basis for TCM research targeting CaMKII and supporting its clinical application in myocardial infarction treatment.

## 7 Molecular docking of drugs and CaMKII

**FIGURE 6:**
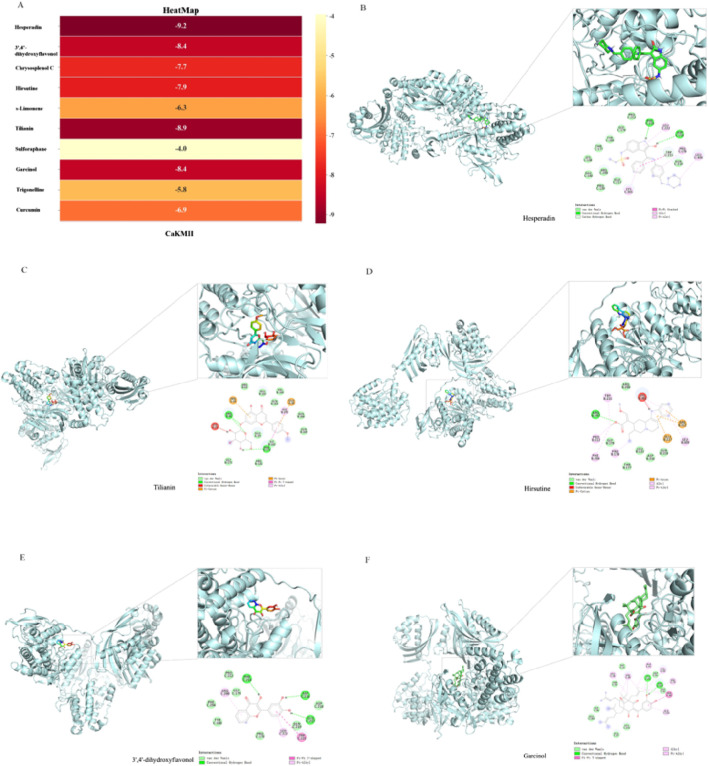
Molecular docking between the natural drug and CaMKII. **(A)** the heatmap of natural drug and CaMKII; **(B)** CaMKII - Hesperadin; **(C)** CaMKII - Tilianin; **(D)** CaMKII - Hirsutine; **(E)** CaMKII - 3'.4′dihydroxyfavonol; **(F)** CaMKII - Garcinol.

Molomplexes exhibited high binding affinity, which was used to assess the binding efficacy between small-molecule drugs and CaMKII, providing insights into the potential therapeutic effects of these drugs on diseases. Negative binding energy indicates that the ligand can bind to the receptor protein. A lower (more negative) binding energy implies a stronger binding affinity between the active metabolite and the target protein. Figure 6A shows the binding energies of CaMKII with each predicted drug, and the docking results of the five natural monomer metabolites with the highest binding energies are also presented (Figures 6B–F). The docking results revealed that most of the complexes exhibited high binding affinity, with an average binding energy of −7.35 kcal/mol. Except for Sulforaphane, the binding energies of other drug monomers were all lower than −5.0 kcal/mol, and 60% of them were lower than −7.0 kcal/mol, indicating good binding ability to the target protein. Among them, Hesperadin showed the best binding efficacy, followed by Tilianin, 3′,4′-dihydroxyflavonol, Garcinol, and Hirsutine, etc. These results suggest that natural drugs possess considerable binding activity towards the CaMKII target.

## 8 Discussion

### 8.1 Context-dependent duality of CaMKII function

CaMKII’s dual role in MIRI—protective or detrimental—depends on the cellular microenvironment and phosphorylation state, complicating phytochemical regulation. Microenvironmentally, ischemia’s hypoxic/acidotic conditions activate CaMKIIδ to induce cardiomyocyte damage *via* apoptotic molecule phosphorylation (Tan et al., 2024), while moderate reperfusion oxidative stress activates CaMKIIγ to promote survival *via* Akt phosphorylation (Yang et al., 2021). Phosphorylation state is equally critical: sustained Thr287 autophosphorylation disrupts mitochondrial function (Munevar et al., 2008; Eilers et al., 2014), while Ser332 phosphorylation inhibits CaMKII to mitigate harm (Shioda and Fukunaga, 2017). Current research rarely accounts for this context dependency, leading to incomplete understanding of phytochemical modulation across MIRI stages. Future studies must dissect how phytochemicals target specific CaMKII subtypes and phosphorylation states to develop precise, stage-specific therapies.

### 8.2 Compositional complexity and mechanistic ambiguities of phytochemicals

Despite the compelling therapeutic potential of phytochemicals in modulating CaMKII to alleviate MIRI, their translation from preclinical research to clinical practice is impeded by interconnected limitations. These challenges span compositional complexity, pharmacokinetic inefficiencies, safety gaps, and the context-dependent duality of CaMKII function—all requiring systematic resolution to advance clinical utility.

Phytochemicals’ intrinsic multi-metabolite and multi-target properties drive intricate yet poorly defined CaMKII regulatory mechanisms. They act *via* synergistic, antagonistic, or additive interactions: synergistically, tanshinone IIA combined with astragaloside IV reduces infarct size (Zhai et al., 2024), astragaloside and puerarin enhance protection *via* PI3K/Akt (An-ran et al., 2023), dihydromyricetin inhibits high glucose-induced CaMKII oxidation to mitigate oxidative stress (Lee et al., 2022), and 3-caffeoyl-4-dicaffeoylquinic acid regulates CaMKII and other kinases to boost nitric oxide production (Lee et al., 2022); antagonistically, glycyrrhetinic acid with aconitine increases LDH release to counteract protection (Yin et al., 2022). However, this complexity creates barriers: different phytochemical classes (e.g., flavonoids, glycosides) may exert opposing effects on CaMKII (Lin and Sun, 2025), and they often regulate interconnected signaling networks (PI3K/Akt/GSK3β, CaMKII/CREB, NF-κB) (Ajzashokouhi et al., 2023; Li et al., 2025), leaving optimal metabolite ratios, action timings, and quantitative interaction mechanisms undefined. While their multi-target nature confers advantages (simultaneous antioxidant, anti-inflammatory effects) (Su et al., 2022a, 2022b), dissecting mechanisms demands systems biology, network pharmacology, and high-throughput screening (Su et al., 2022b), with future research needing to isolate key metabolites, apply mathematical models (e.g., response surface methodology), and use proteomics to clarify CaMKII subtype selectivity and downstream regulation (ERK1/2, NF-κB) (House and Singer, 2008).

### 8.3 Pharmacokinetic hurdles limiting therapeutic efficacy

Pharmacokinetic inefficiencies are a major bottleneck for CaMKII-targeting phytochemicals. Poor bioavailability stems from low intestinal permeability and hepatic first-pass metabolism—baicalin and tanshinone IIA, for example, reach sub-therapeutic myocardial concentrations (Semalty et al., 2010; Estrela et al., 2017) and multi-metabolite systems exhibit variable absorption, distribution, metabolism, and excretion (ADME) rates. Dose determination relies on *in vitro* or rodent models, lacking unified human conversion standards and ignoring comorbidities’ impact on drug metabolism. Promising solutions include nano-drug delivery systems (e.g., liposomes) that boost tanshinone IIA bioavailability 2–3-fold (Hu et al., 2010; Okur et al., 2021), and establishing an “in vitro-in vivo-clinical” dose model integrated with pharmacokinetic-pharmacodynamic (PK/PD) analysis to define the therapeutic window (Titze et al., 2017; van Os and Zeitlinger, 2021).

### 8.4 Safety gaps and clinical translation barriers

At present, clinical translation faces a major bottleneck due to a lack of safety data and limitations in large-scale clinical validation, halting progress. Most preclinical findings, like PNS reducing infarct size in mouse AMI models *via* CaMKII phosphorylation (Wang et al., 2021a), lack human confirmation. And without multi-center trials, validating efficacy across diverse populations is missing. Safety assessment has glaring gaps. Most studies observe for under 4 weeks, preventing cumulative toxicity evaluation, e.g., high-dose curcumin causing excessive cardiomyocyte autophagy (Skinner et al., 2018). Monitoring CaMKII subtype balance, vital as its disruption triggers adverse reactions (House and Singer, 2008), is rare. Systematic evaluations of drug-drug interactions and dose-dependent toxicity are also underdeveloped.

Future research should merge long-term toxicology, mechanistic safety tests, and well-planned clinical trials to verify drug efficacy and safety in humans. Substances from plants, algae, fungi, lichens, or animals bring unique challenges. These extracts are multi-component mixtures with non-single-target activities (Heinrich et al., 2022). Their composition varies with preparation methods and plant materials, undermining reproducibility and result interpretation in pharmacological, toxicological, and clinical research.

### 8.5 Insufficient number of studies and superficial mechanistic research

Current research on natural drug modulation of CaMKII for treating myocardial ischemia-reperfusion injury has limitations. Experimentally, relevant articles are scarce, limiting data accumulation. Mechanistically, many studies do not clarify the direct way natural drugs regulate CaMKII. They also lack comparison with known inhibitors like KN - 93 and AIP, hindering efficacy evaluation. Clinically, there are no trials, so the real - world safety and effectiveness of natural drugs in humans remain unknown. These limitations call for more in - depth experimental and clinical research.

## 9 Conclusion and future perspectives

Due to various pharmacological reasons, natural products and dietary metabolites have recently gained increasing popularity. This has emerged as a viable approach for managing and treating MIRI and mitigating its consequences. This review, for the first time, systematically investigates how natural metabolites targeting CaMKII can be used to treat MIRI. The results indicate that flavonoids, alkaloids, glycosides, terpenes, polyphenol analogs, and traditional Chinese medicines contribute to regulating the expression of CaMKII. Indicators of cardiomyocyte apoptosis and inflammation are improved. To varying degrees, the pathogenic processes of MIRI, including mitochondrial dysfunction, calcium overload, and inflammatory responses, are also suppressed.

A comprehensive review of current research reveals that the CaMKII signaling pathway plays a central role in the pathogenesis of MIRI. It serves not only as a key regulatory node in the pathological process but also as an ideal target for drug intervention. This understanding provides an important direction for exploring more effective treatment strategies, especially showing broad prospects in the research and application of natural drugs.

Multiple studies have clearly demonstrated that CaMKII directly participates in the apoptosis and necrosis processes of cardiomyocytes by regulating calcium homeostasis, oxidative stress responses, and inflammatory responses in these cells. Natural drugs, with their multi-target and multi-mechanism characteristics, can synergistically regulate CaMKII and its downstream signaling pathways, thereby achieving comprehensive effects of antioxidant, anti-inflammatory, and anti-apoptotic actions. This multi-dimensional intervention approach offers advantages over single-target drugs, as it can more effectively alleviate the multiple injuries caused by MIRI and enhance myocardial protection.
